# Evaluating the efficiency of carbon utilisation via bioenergetics between biological aerobic and denitrifying phosphorus removal systems

**DOI:** 10.1371/journal.pone.0187007

**Published:** 2017-10-24

**Authors:** Zhan Jin, Fangying Ji, Yin He, Min Zhao, Xuan Xu, Xiang-yong Zheng

**Affiliations:** 1 College of Life and Environmental Science, Wenzhou University, Wenzhou, China; 2 Key Laboratory of Three Gorges Reservoir Region’s Eco-Environment, Ministry of Education, Chongqing, China; The Education University of Hong Kong, HONG KONG

## Abstract

There are two biological systems available for removing phosphorus from waste water, conventional phosphorus removal (CPR) and denitrifying phosphorus removal (DPR) systems, and each is characterized by the type of sludge used in the process. In this study, we compared the characteristics associated with the efficiency of carbon utilization between CPR and DPR sludge using acetate as a carbon source. For DPR sludge, the heat emitted during the phosphorus release and phosphorus uptake processes were 45.79 kJ/mol e^-^ and 84.09 kJ/mol e^-^, respectively. These values were about 2 fold higher than the corresponding values obtained for CPR sludge, suggesting that much of the energy obtained from the carbon source was emitted as heat. Further study revealed a smaller microbial mass within the DPR sludge compared to CPR sludge, as shown by a lower sludge yield coefficient (0.05 gVSS/g COD versus 0.36 gVSS/g COD), a result that was due to the lower energy capturing efficiency of DPR sludge according to bioenergetic analysis. Although the efficiency of anoxic phosphorus removal was only 39% the efficiency of aerobic phosphorus removal, the consumption of carbon by DPR sludge was reduced by 27.8% compared to CPR sludge through the coupling of denitrification with dephosphatation.

## Introduction

Nitrogen (N) and phosphorus (P) are the key nutrients that cause eutrophication in waterways. In conventional biological wastewater treatment, P removal is achieved through enhanced biological phosphorous removal under alternating anaerobic–aerobic conditions, which is known as conventional phosphorus removal (CPR). The removal of nitrogen is accomplished by a two-stage treatment process, which consists of nitrification and denitrification. Both P removal and N removal require a carbon source. However, carbon is often present in low level in sewage water with low carbon/nitrogen ratio. In denitrifying phosphorus removal (DPR), nitrate is used as an electron acceptor, which allows denitrification and P uptake to occur simultaneously using the same organic carbon source [1,2]. In this way, DPR offers an appropriate solution to the problems associated with the limitation of chemical oxygen demand (COD) that leads to the low N and P removal efficiency [3,4,5]. Kuba [6] found that by coupling P removal with N removal in a so-called two-sludge system, the required COD, aeration cost, and sludge production can be reduced as much as 50%, 30% and 50%, respectively. Another study has shown that a modified two-sludge AAO-biological contact oxidation (BCO) system, which incorporates DPR into the process, can reduce the consumption of carbon by about 30–35% compared to the traditional biological nutrient removal (BNR) systems [7]. However, other studies have suggested that P uptake under denitrifying conditions requires 1.67 times more COD than operation under aerobic conditions in order to achieve the same efficiency for phosphorous removal. because the energy production efficiency from carbon oxidation using nitrate as an electron acceptor is about 40% lower than that obtained with oxygen as an electron acceptor, a phenomenon that is illustrated by the Delft model, which is based on biochemical knowledge and stoichiometry [8]. Thus, the possible advantages of DPR in sewage water with low C/N ratio have yet to be fully investigated in detail.

According to the Delft model, the whole energy pathway roughly includes energy for work (biological activities such as phosphorus transport, etc. and cell proliferation such as poly-P synthesis, glycogen regeneration, etc.) and heat output associated with cell maintenance during the P uptake phase [8]. When more heat is released into the environment, less energy is available for other cellular activities. Therefore, the metabolic characteristics of the microorganisms in the sludge can be analyzed from the pattern of heat energy released into the environment. The main objective of this study was to examine the metabolic characteristics of DPR sludge via microcalorimetry, a thermal analysis technique that allows analysis to be performed directly on a test substance, regardless of its homogeneous or heterogeneous nature. Microcalorimetry permits online bioactivity screening and can provide a lot of important information on the process of cell growth. It allows a continuous measurement of heat production to describe the growth process without disturbing the normal activity of the bio-system. At the same time, it does not require a homogeneous system and continuous, in situ, real time, quantitative detection can be realized so as to obtain abundant thermo-kinetic/dynamic information about the microbial metabolism progress [9,10]. Currently, microcalorimetry is extensively used in life science research to study a number of biological processes, such as cellular growth, development, metabolism and energy transfer [11,12,13]. However, it has not been applied to the study of biochemical mechanisms involved in wastewater treatment process.

In this study, microcalorimetry was used to monitor the production of heat by DPR sludge during the stages of phosphorus uptake and release in low C/N ratio sewage water. In addition, the mount of ATP produced per electron pair (δ or ATP/NADH ratio) and the energy for the transport of phosphate (ε or P/NADH2 ratio) were calculated from the bioenergetics perspective to determine the efficiency of carbon utilization.

## Materials and methods

### Microcalorimetry

The RD496-2000 microcalorimeter (developed jointly by the China Academy of Engineering Physics and Mianyang CP Thermal Analysis Instrument Co., Ltd.) used in this study has a sensitivity of 50–70 μv/mW, a temperature-controlled precision of ±0.001°C/h, a calorimetric accuracy of 1%, and an output range of 2 μW to 1W. The details of its construction have been described previously [14].

### Experimental materials

Sludge samples were collected from two different biological phosphorus removal systems, respectively. Conventional phosphorus removal (CPR) sludge was taken from the external recycling process of aerobic sludge in an SBR system (ERP-SBR) [15], whereas DPR sludge was taken from the denitrifying phosphorus removal system with anaerobic/anoxic and nitrifying of phostrip process in SBR (A2N-P-SBR)[16].

The medium used in the microcalorimtry analysis consisted of 1 g /L sodium acetate standard solution (about 744.2 mg COD/L) and 0.6 mol/L trace elements. Each liter of trace element solution contained 1.5 g FeCl_3_·6H_2_O, 0.15 g H_3_BO_3,_ 0.03 g CuSO_4_·5H_2_O, 0.03 g KI, 0.06 g Na_2_MoO_4_·2H_2_O, 0.12 g MnCl_2_·4H_2_O, 0.12 g ZnSO_4_·7H_2_O, and 0.15 g CoCl_2_·2H_2_O. The nitrate solution used had a concentration of 200 g/L KNO_3_. This concentration was selected to provide an excess amount of nitrate in the anoxic phase. Pure argon was used to provide an anaerobic condition, whereas air consisting of 10% oxygen and 90% argon was used to provide an aerobic condition.

### Experimental set-up

Sludge samples (2 L each) were collected separately from both the aerobic phase of ERP-SBR process and the A_2_N-P-SBR process after the pollutant removal rate had stabilized. The samples were first concentrated to allow their mixed liquor suspended solids (MLSS) to reach 12g/L, and 400 ml of each sample was removed, washed with distilled water followed by centrifugation. After removing the bulk of the supernatant, the residual sludge was placed in a 500-ml beaker. The sludge was aerated for 30 min to clear the remaining carbon and then placed in a 28°C water bath. After the treatment, the sludge sample was placed in a furnace inside a microcalorimeter.

Anaerobic process: First, 1.8 ml of the pretreated sludge was placed in the test furnace sample cell of the microcalorimeter, and 0.2 ml medium was placed in the kinescope liner in the sample cell. The sludge and medium were then transferred to the sealed testing pool. The sample cell and the kinescope of the reference pool contained 1.8 ml and 0.2 ml distilled water, respectively. After all the components were placed in the furnace, argon was introduced into the furnace at a flow rate of 4 ml/min, and the furnace body temperature was set to 28°C. The heat change in the furnace was monitored by the computer and shown as a plot. When the electric potential of the plot was stable (around 2 h), the kinescopes in the testing pool and the reference pool were pierced simultaneously by the aeration pipe, allowing the sludge and the medium to mix. After that, the anaerobic phase began, and the reaction ended when the electric potential leveled off again.

Anoxic/post-aerobic process: First, 0.2 ml medium was added to the sample cell already containing 1.8 ml sludge, and argon was then introduced into the furnace at a flow rate of 4 ml/min. The furnace temperature was set to 28°C for strict anaerobic reaction. When the electric potential became stabilized, the kinescope containing 0.2 ml of KNO_3_ solution was placed in the sample cell. Distilled water was used as the reference. When the electric potential leveled off after a period of fluctuation, the aeration pipe pierced the two kinescopes simultaneously, and nitrate was mixed with the above solutions, which had been through an anaerobic process, to start the anoxic denitrification phase. The supplied argon gas was turned off at the end of the anaerobic process (as indicated by the electric potential reaching a plateau) and oxygen was then introduced into the furnace to initiate the aerobic phase. The post-aeration phase continued until the electric potential curve leveled off again.

Each of the above experiments was performed three times. Paired-samples t-test (95% confidence interval) was used to determinate the statistical significance of the difference in the ratios of heat output between the two sludge samples and between different phases using the SPSS (Statistical Product and Service Solutions, Version 7) software (P <0.05 indicated statistical significance).

### Energy calculations

The energy requirement for cell growth depends on the specific carbon and nitrogen sources available for cell growth. For heterotrophic bacteria, different carbon sources will produce different amounts of energy. If pyruvate is an organic intermediate of cell synthesis, energy production or consumption will depend on the relationship between the final products and the free energy of pyruvate. Taking pyruvate as an organic intermediate of carbon in the cell, the energy for cell synthesis can be estimated by the following equation [17]:

$$\Delta G_{S}=\frac{\Delta G_{P}}{k^{m}}+\Delta G_{c}+\frac{\Delta G_{N}}{K}$$ (1)

Where, △G_S_ represents the free energy of 1 electron equivalent (e^-^eq) of carbon source converted to cell substances; △G_P_ represents the free energy of 1e^-^eq of carbon source converted to the pyruvate intermediary; K is the captured ratio in the energy transformation; m equals +1 when △G_P_ is positive or –1 when △G_P_ is negative; △G_c_ represents the free energy of 1e^-^eq of pyruvate intermediary converted to 1 e^-^eq cell; and △G_N_ is the free energy of the reduction of nitrogen to ammonia by 1 e^-^eq cell and equals 17.46 kJ/mol e^-^ when NO_3_^—^N is the nitrogen source. △G_P_ (sodium acetate converted to pyruvate) was +8.12 kJ/mol e^-^, which was positive, and therefore, m = +1, and △G_c_ was 31.41 kJ/mol e^-^. Thus, when NaAc was the carbon source, the free energy for the transformation from 1 electron equivalent (e^-^eq) of carbon source to cell substances is:

$$\Delta G_{S}=\frac{8.12}{k^{1.0}}+31.41+\frac{17.46}{K}$$ (2)

A portion of electron donor (carbon source) captured by the cell is used for cell synthesis, and another portion is oxidized to provide energy for cell synthesis. The energy balance is shown in the following equation, where the left-hand side of the equation is the available energy and the right-hand side is the energy for cell growth.

$$K\Delta G_{R}(\frac{f_{e}}{f_{s}})=-\Delta G_{S}$$ (3)

$$f_{e}+f_{s}=1$$ (4)

In these equations, K is the captured energy ratio; △G_R_ is the energy released in the redox reaction (kJ/mol e^-^); *f*e is the ratio of electron donors (captured by the organisms) for available energy; and *f*s is the ratio of electron donors (captured by the organisms) for synthesis.

Since NADH_2_ is the equivalent of 2e^-^, δ (ATP/NADH_2_ ratio, mmol/mmol) and ε (P-mol/mol NADH_2_) were calculated from the following equation.

$$\delta =\frac{K\times \Delta G_{R}\times f_{e}\times 2}{30.54}$$ (5)

ε=3.8×δ (6)

## Results and discussion

### Thermodynamic analysis of CPR sludge and DPR sludge

**Fig 1:**
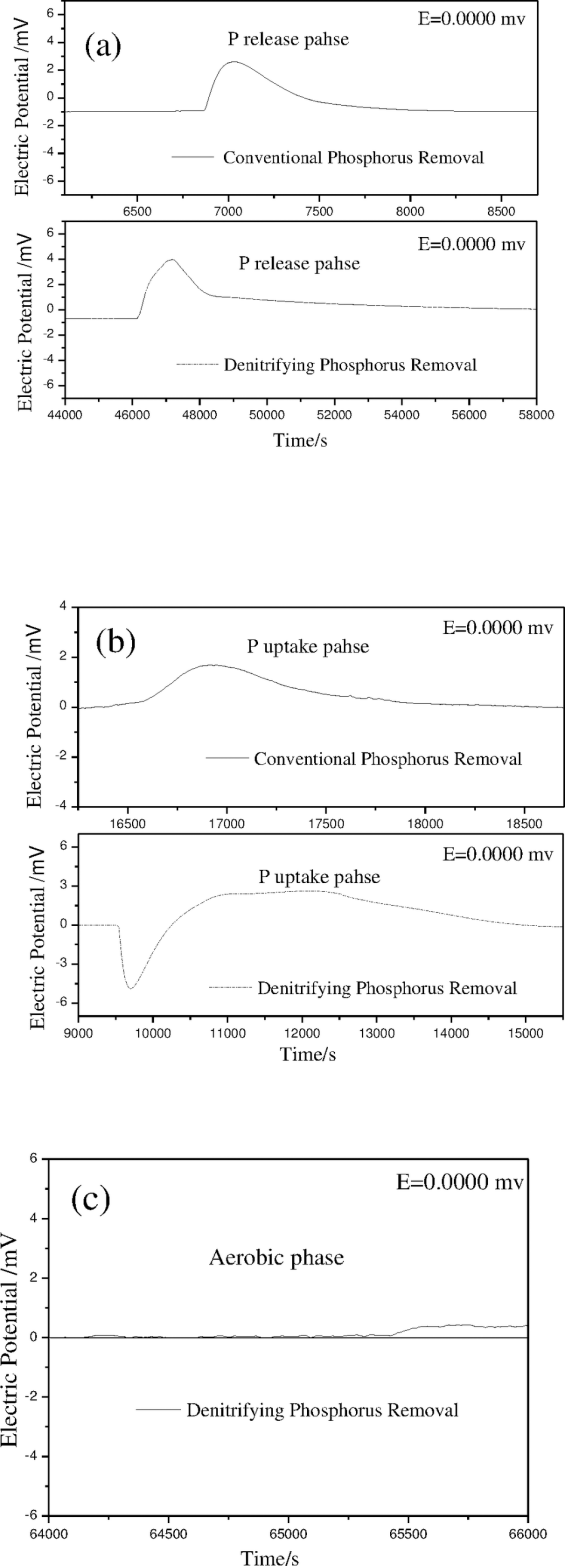
Heat generation by CPR sludge and DPR sludge in different phases. (a: heat variation processes of the two sludge samples during P release phase; b: heat variation of the two sludge samples during P uptake phase; c: heat variation processes of DPR sludge during aerobic phase).

The heat change for the CPR sludge associated with the release of phosphorous via anaerobic and aerobic processes was determined by microcalorimetry. The heat change of CPR sludge in anaerobic P release and excess aerobic P uptake was determined by microcalorimetry. Poly-P (poly- phosphorus) decomposition and PHB (poly-β-hydroxybutyrate) synthesis occurring in the anaerobic phase were clearly exothermic processes (Fig 1A). Furthermore, the oxidative decomposition of PHB and poly-P synthesis occurred in the aerobic phase were also exothermic process. In the anaerobic phase, the exothermic reaction rate increased rapidly, reaching to an exothermic peak within 5–12 min, and then gradually decreased. The whole anaerobic exothermic process lasted only for 30–44 min, producing a total heat energy output of 20.59 kJ/mol e^-^ (Table 1). These results illustrated that after pretreatment by aeration, residual carbon source had been completely consumed, and the bacteria were under starvation condition. When the medium containing sodium acetate as the carbon source was added to the system, the sludge absorbed acetic acid and released heat rapidly through the hydrolysis of poly-P. The total heat output in the aerobic phase was 35.08 kJ/mol e^-^(Table 1), which was greater than that produced in the anaerobic phase (20.59 kJ/mol e^-^).

**Table 1:** Heat output produced by the CPR and DPR sludges (kJ/mol e-) during P release and uptake.

	H p-release (±SD)	H p-uptake (±SD)
CPR sludge	20.59±3.04	35.08±12.44
DPR sludge	45.79±3.41	84.09±9.46

H—heat outputs; SD—standard deviation.

Heat changes produced by the DPR sludge during the anaerobic P release, anoxic denitrifying P uptake, and the post-aeration phases are shown in Fig 1B. The total heat output produced by the DPR sludge during the anaerobic phase was 45.79 kJ/mol e^-^ (Table 1). At the beginning of the anoxic phase, the heat absorption was about 32.85 kJ/mol e^-^, and more heat was released during the later period, increasing the total heat output to 84.09 kJ/mol e^-^ (Table 1). Unlike the aerobic uptake of phosphorous, the uptake of phosphorous by DPR sludge was first endothermic, but then became exothermic. This might be due to the energy expended during the transport of nitrates into the cells [18]. There are four steps in the process of denitrification (NO_3_^—^N→NO_2_^—^N→NO→N_2_O→N_2_) when nitrate is used as an electron acceptor, with each step resulting in the production of ATP [19]. Therefore, the heat output of the system increased and was greater than the heat uptake as the denitrifying P uptake reaction progressed. The net heat output of the total anoxic phase was 51.44 kJ/mol e^-^. The heat change that occurred during the post-aeration phase of the DPR system was almost negligible (Fig 1), indicating that the external carbon source was completely consumed and PHB stored in the DPR sludge during the anaerobic phase was fully used.

The ratios of heat output of P release to P uptake were not statistically different (P = 0.589) between the two sludge samples, indicating a similar phosphorous metabolism occurring in DPR and CPR sludges (Table 2), consistent with the result of a previous study [20]. Further analysis found that both DRP and CRP sludges also had similar heat output ratios during the P release phase and P uptake phase (P = 0.515), approximately 40% (Table 2). These results indicated that the heat output from the CPR sludge was about 40% that of DPR sludge, in both P release and P uptake phases. When the solid retention time (SRT) of the system and the nutrients used were the same for the two sludges, CRP sludge had a higher capacity to capture the energy than DPR sludge, which used nitrates as an electron acceptor. For this reason, a large amount of carbon consumed was converted into cell materials in the case of CPR sludge. This suggested that with DPR sludge, a greater carbon consumption occurred during the process of P removal was not only because of low energy production efficiency, but may also be due to lower energy capturing efficiency.

**Table 2:** Differences in heat output between CPR and DPR sludges and between aerobic and anaerobic phases.

		ratio	P
CPR sludge	H_p-release_/H_p-uptake_	0.59±0.11	0.589
DPR sludge		0.55±0.02	
p-release phase	H_CPR sludge_/H_DPR sludge_	0.45±0.04	0.515
p-uptake phase		0.41±0.09	

H: heat outputs; P: P value of paired-samples t test.

### Thermodynamic analysis of DPR sludge

After the carbon source (acetic acid) entered the system, energy was produced by the CPR sludge. One portion of the energy was captured by the cells, and the other was released into the environment. The energy produced by the complete oxidative decomposition of acetate can be calculated according to the standard free energies of known reactants and products in the half-reaction. Thus, the half-reaction free energy change in the biological system can be expressed as:

$$\frac{1}{8}CH_{3}COO^{-}+\frac{1}{4}O_{2}\to \frac{1}{8}CO_{2}+\frac{1}{8}HCO_{3}^{-}+\frac{1}{8}H_{2}O-105.82kJ$$

Acetate produces a ΔG_R_ = –105.82 kJ/mol e^-^ when oxidation is completed. The heat output in the anaerobic phase was derived from the decomposition of poly-P in the CPR sludge, and thus, in the experiment, the heat output from the oxidation of the sodium acetate should only consist of the heat output of the aerobic phase (35.08 kJ/mol e^-^), meaning that the energy captured by cells should be 70.74 kJ/mol e^-^. One portion of this effective utilized carbon source was used for cell synthesis, whereas the other portion was oxidized to provide energy for the biological activities. According to Eq (2), K was found to be 0.67, and therefore, the free energy of 1 electron equivalent (e^-^eq) of carbon source converted to cell substances (ΔG_S_) was 69.58 kJ/mol e^-^ when acetic acid was the carbon source. Combined with Eq (3), the oxidized molar ratio (*f*e) of carbon source was calculated to be 0.49. The ratio for cell growth (*f*s) was 0.51 g COD (cell)/g COD. As for the organisms (C_5_H_7_NO_2_), 1 g of cells is equal to 1.42 g COD, and thus, the cell yield (Y) obtained from the CPR sludge was 0.36 g VSS/g COD.

Cell yield of DPR sludge: In this study, acetic acid was the carbon source and nitrate was the electron acceptors. Thus, the half-reaction free energy change in the biological system could be expressed as:

$$\frac{1}{5}NO_{3}^{-}+\frac{1}{8}CH_{3}COO^{-}+\frac{1}{5}H^{+}\to \frac{1}{10}N_{2}+\frac{9}{40}H_{2}O+\frac{1}{8}HCO_{3}^{-}-99.35kJ$$

Theoretically, the exhaustive reaction could produce 99.35 kJ/mol e^-^ energy, and therefore, with nitrates as electron acceptors, the theoretical heat output per unit electron with exhaustive oxidation of sodium acetate would be slightly lower than that of the CPR sludge with free oxygen as the electron acceptor. Just as in the case of CPR sludge, the heat output in the anaerobic phase was not included in the total heat output of the sodium acetate reaction. The heat output by DPR sludge during the anoxic phase was 84.09 kJ/mol e^-^ as measured by microcalorimetry, and the energy from the carbon source captured by cells was 15.26 kJ/mol e^-^. In the DPR system, K was 0.15, and ΔG_S_ was 197.5 kJ/mol e^-^ in the DPR system. Therefore, *f*e = 0.93 and *f*s = 0.07 g COD (cell)/g COD, and the cell yield was calculated to be 0.05 g VSS/g COD, which was obviously lower than that of CPR sludge (0.36 g VSS/g COD).

Thermodynamic analysis showed that when the concentrations of carbon sources and MLSS were the same, more acetic acid was released into the environment in the form of heat and less was used for synthesis of cell materials by the DPR sludge during the anaerobic/anoxic phases. Due to the lack of available carbon source in the post-aeration phase, the heat output of the oxidation reaction was negligible. The bioenergetics calculation results suggested that the main reason for the low sludge yield of DPR sludge could be due to the lower energy capturing efficiency of the sludge compared to that of CPR sludge.

### Carbon source utilization efficiency of DPR sludge

To further investigate the differences in carbon source utilization efficiency between CPR sludge and DPR sludge, the energy for the transport of phosphate (ε) during the anoxic phase in the case of DPR sludge, or the aerobic phase in the case of CPR sludge, was calculated from the bioenergetics results described above and the captured energy obtained from computer analysis. With sodium acetate as the carbon source, the value of ε_DPR_ determined from the anoxic phase (3.4 P-mol/mol NADH_2_) was only 39% of the value of ε_CPR_ obtained from the aerobic phase (8.7 P-mol/mol NADH_2_) (Table 3). In other words, the consumption of carbon required for the anoxic absorption of the same amount of P by DPR sludge was 2.6 times the amount required by the CPR sludge. For each electron equivalent required for the removal of P by the CPR sludge, the DPR sludge would need 2.6 times more, but at the same time, it also removed 2.6 electron equivalents from the nitrogen in nitrate.

**Table 3:** Energy consumed by CPR and DPR sludges in the synthesis process.

	K△G_R_(kJ/mol e-)	*f*e	E	δ	ε
CPR sludge	70.74	0.49	34.66	2.27	8.7
DPR sludge	15.26	0.93	14.19	0.9	3.4

E: Available energy captured by microorganisms.

Thus, DPR sludge would only require 2.6 e^-^eq of carbon source while CPR sludge would require 3.6 e^-^eq of carbon source to remove the same amount of N and P. It was clear that compared to CPR sludge, DPR sludge consumed 27.8% less carbon in the process of N and P removal, which translated to a 50-% reduction in the amount of COD required, and this was obviously lower than that reported in previous study [6]. In addition to the integration of denitrification and dephosphatation, the replacement of aerobic P uptake with anoxic P uptake may be another reason for the lower carbon consumption by DPR sludge, which effectively decreased the COD aerobic loss of the system.

## Conclusions

Microcalorimetry analysis of CPR and DPR sludge showed that the heat outputs produced by the DPR sludge during the processes of P release and P uptake were two fold higher than those produced by the CPR sludge for the corresponding processes. Bioenergetics-based analysis revealed a lower energy capturing capacity for the DPR sludge, and this was the main reason for the low sludge yield of DPR sludge. The study also found that the phosphorus removal efficiency of the DPR sludge was only 40.9% that of the CPR sludge, but by coupling denitrification to dephosphatation, DPR sludge managed to use less carbon.

## Supporting information

S1 File This includes Excel worksheets 1–4. Worksheets 1. Data of Heat generation by DPR sludge in aerobic phase. Worksheets 2. Data of Heat generation by CPR sludge and DPR sludge in P release phase. Worksheets 3. Data of Heat generation by CPR sludge and DPR sludge in P uptake phase. Worksheets 4. Heat output in different phases of the two sludge samples.
(DOC)

## References

[1] Lotter LH. The role of bacterial phosphate metabolism in enhanced phosphorus removal from the activated sludge process. Water Sci Tech. 1985; 17: 127–138.

[2] Liu S., Li J. Accumulation and isolation of simultaneous denitrifying polyphosphate-accumulating organisms in an improved sequencing batch reactor system at low temperature. Int Biodeterior Biodegrad. 2015; 100: 140–148.

[3] Hascoet M. C., Florentz M. Infuence of Nitrates on Biological phosphorus removal nutrient wastewater. Wat SA. 1985; 11(1): 1–8.

[4] Bassin J.P., Kleerebezem R., Dezotti M., van Loosdrecht M.C. Simultaneous nitrogen and phosphate removal in aerobic granular sludge reactors operated at different temperatures. Water Res. 2012; 46(12): 3805–3816. doi: 10.1016/j.watres.2012.04.015 22591819 10.1016/j.watres.2012.04.015

[5] Kim J.M., Lee H.J., Lee D.S., Jeon C.O. Characterization of the denitrification-associated phosphorus uptake properties of “Candidatus Accumulibacter phosphatis” clades in sludge subjected to enhanced biological phosphorus removal. Appl. Environ. Microbiol. 2013; 79 (6): 1969–1979. doi: 10.1128/AEM.03464-12 23335771 10.1128/AEM.03464-12 PMC3592233

[6] Kuba T., Van Loosdrecht M.C.M., Heijnen J.J. Phosphorus and nitrogen removal with minimal COD requirement by integration of denitrifying dephosphatation and nitrification in a two-sludge system. Water Res. 1996; 30(7): 1702–1710.

[7] Zhang M., Peng Y, Wang C., Wang C., Zhao W., Zeng W. Optimization denitrifying phosphorus removal at different hydraulic retention times in a novel Anaerobic Anoxic Oxic-Biological Contact Oxidation process. Biochem Eng J. 2015; 106: 26–36.

[8] Kuba T., Murnleitner E., Van Loosdrecht M.C.M., Heijnen J.J.A Metabolic Model for Biological Phosphorus Removal by Denitrifying Organisms. Biotechnol. Bioeng. 1996; 52(6): 685–695. doi: 10.1002/(SICI)1097-0290(19961220)52:6<685::AID-BIT6>3.0.CO;2-K 18629947 10.1002/(SICI)1097-0290(19961220)52:6<685::AID-BIT6>3.0.CO;2-K

[9] Critter S. A. M., Freitas S. S., Airoldi C. Microbial biomass and microcalorimetric methods in tropical soils. Thermochim Acta. 2002; 394(1): 145–154.

[10] Liu L., Lin G.M., Shao W. Application of microcalorimetry in the research of microorganism and cell. Pharm. Biotechnol. 2010; 137(1): 7753–7767.

[11] Debord J., Laubarie C., Dantoine T. Microcalorimetric study of the inhibition of butyrylcholinesterase by carbamates. Anal Biochem. 2008; 373(2): 97–101. 10.1016/j.ab.2007.09.023 17950687

[12] Cesar G. L., Elizabeth C. M., Nora Ponce de L. R., Francisco V. A., Leticia B. L., Victor M. G. P. Microcalorimetric measurement of Trichoderma spp. growth at different temperatures. Thermochim Acta. 2010; 509(1–2): 40–45.

[13] Kong W. J., Wang J.B., Xing X.Y., Jin C., Xiao X.H., Zhao Y.L. et al Screening for novel antibacterial agents based on the activities of compounds on metabolism of Escherichia coli: A microcalorimetric study. J. Hazard. Mater. 2010; 185(1): 346–352. doi: 10.1016/j.jhazmat.2010.09.040 20926184 10.1016/j.jhazmat.2010.09.040

[14] Ji M., Liu M.Y., Gao S.L., Shi Q.Z. A new microcalorimeter for measuring thermal effects. Instrum. Sci. Technol. 2001; 29(29): 53–57.

[15] Ji F. Y., Xiao T. Y., Gu Y. Influencing factors of biological nitrogen removal in the bypass phosphorus-removal ERP-SBR. Tech. Eq. Environ. Pollut. Control. 2006; 7(7): 20–24.

[16] He Y. Effect of SRT on Denitrifying Phosphorus Removal System with Side-stream Phosphorus Removal. M. Eng. Thesis, Chongqing university. 2011.

[17] Metcalf & Eddy Inc.Wastewater Engineering: Treatment and Reuse 4th ed. Beijing: Chemical Industry Press; 2004.

[18] Zheng P. New theory and biotechnology for nitrogen removal Beijing: Sicence Press 2004.

[19] Wang SY, Sun H.W., Yang Q., Peng Y.Z. Biochemical Reaction Mechanism and Kinetics of Denitrification. Chin. J. Appl. Environ. Biol. 2008; 14: 4.

[20] Kuba T, Smolders G., van Loosdrecht M.C.M., Heijnen J.J. Biological phosphorus removal from wastewater by anaerobic-anoxic sequencing batch reactor. Water Sci Tech. 1993; 27: 11.

