# Supplementary material for: Evaluating the efficiency of carbon utilisation via bioenergetics between biological aerobic and denitrifying phosphorus removal systems
Source: PLoS One. 2017 Oct 24;12(10):e0187007. doi: 10.1371/journal.pone.0187007 (PMC5655452; doi:10.1371/journal.pone.0187007)
Supplement: S1 File — Worksheets 1. Data of Heat generation by DPR sludge in aerobic phase. Worksheets 2. Data of Heat generation by CPR sludge and DPR sludge in P release phase. Worksheets 3. Data of Heat generation by CPR sludge and DPR sludge in P uptake phase. Worksheets 4. Heat output in different phases of the two sludge samples. (DOC) [file pone.0187007.s001.doc]

Supplementary Material

**1. The operation mode of these two systems**

The external recycling process of aerobic sludge used in our SBR system (ERP-SBR) consisted of three reactors: SBR reactor, enhanced anaerobic phosphorus release tank and chemical phosphorus removal tank. The operation mode of the SBR reactor was anaerobic 3.5h / aerobic 4.0h / anoxic 2.5h / aeration 1.0h / sedimentation and drainage 1.0 h. The aerobic sludge in the SBR reactor was discharged into the enhanced anaerobic phosphorus release tank at the end of the cycle and the phosphorus released sludge was pumped back to the SBR reactor during the aerobic phosphorus absorption process. Meanwhile, the phosphorus rich supernatant of the anaerobic tank was introduced into the chemical phosphorus removal tank, and then the supernatant was fed into the influent and allowed to flow back into the SBR reactor.

**
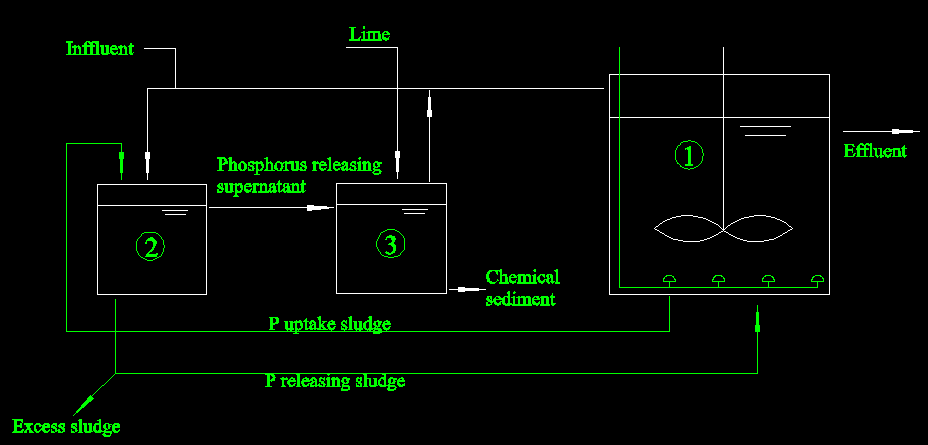
**

Fig.1. Flow chart showing the design of the ERP-SBR system and the process it operates (1: SBR reactor; 2: Enhanced anaerobic phosphorus release tank; 3: Chemical phosphorus removal tank)

The denitrifying phosphorus removal system with anaerobic/anoxic and nitrifying of phostrip process in SBR (A_2_N-P-SBR) consisted of two SBRs. One SBR was operated for denitrifying dephosphatation-SBR(I), whereas the other SBR was operated under aerobic conditions for nitrification-SBR(II). In this combined system (called A_2_N-P-SBR), DPB and nitrifiers were completely separated, and only the supernatant was exchanged between the two SBRs. The design of the A_2_N-P-SBR system and its associated process are shown in Figure 4.

SBR (I): Anaerobic 90min/ Sedimentation and supernatant efflux to SBR (II) 80 min/ Anoxic 190 min/ Post-aerobic 60 min/ Sedimentation and drainage 60 min.

SBR (II): Aerobic 260 min/ idle 200 min/ supernatant backflow to SBR (I) 20 min.


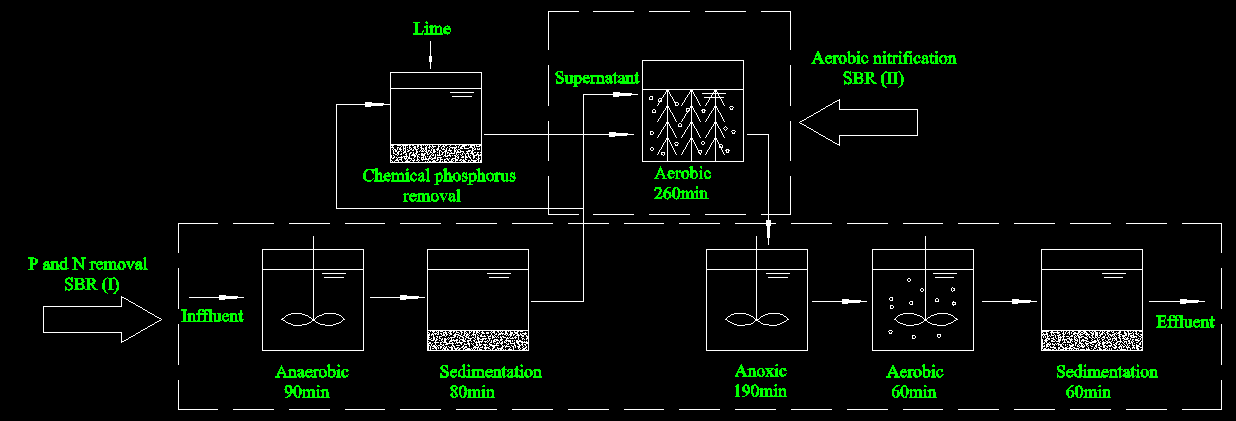


Fig.2. Flow chart showing the design of the A_2_N-P-SBR system and its associated process.

**2. Periodic variation P and NO_3_^-^-N of these two systems during the P uptake process**

The P periodic variation (as well as nitrate as the electron acceptor in the DPR system) of the two different biological phosphorus removal systems during the P uptake process are shown in Figure 1-3. The average amount of COD absorbed by the bacteria in the anaerobic phase is shown in Table 1. The P uptake of the CPR sludge was 45.9 mg/L in the aerobic phase, whereas the DPR P uptake of the DPR sludge was 7.2 mg/L due to the limitation of nitrogen concentration (15.6 mg/L).

Table 1. COD concentration in the influent and the average absorption amount of COD by the two different bacteria in the anaerobic phase

|  | Influent | The average absorption amount of COD |
| --- | --- | --- |
| PAO (mg/L) | 266.83 | 199.04 |
| DPAO (mg/L) | 217.24 | 220.41 |

Fig. 3. P periodic variation of the external recycling process of aerobic sludge in an SBR system (ERP-SBR) for CPR sludge.

Fig. 4. P periodic variation of the denitrifying phosphorus removal system with anaerobic/anoxic and nitrifying of phostrip process in SBR (A2N-P-SBR) for DPR sludge

Fig. 5. NO_3_^-^-N periodic variation of the denitrifying phosphorus removal system with anaerobic/anoxic and nitrifying of phostrip process in SBR (A2N-P-SBR) for DPR sludge

**2. The difference between PAOs and DPAOs**

Table 2 Summary of the difference between the DPAO and PAO

| PAO | DPAOs | Parameter | Unit | Ref. |
| --- | --- | --- | --- | --- |
| 1.85 | 0.9 | δ | mol ATP/ mol NADH_2_ | [[[1]](#endnote-2),[[2]](#endnote-3)] |
| 7 | 3.5 | ε | P-mol/mol NADH_2_ |  |
| 4×10^3^ | 2.5×10^3^ | m_ATP_ | mol P/mol C • h | [[[3]](#endnote-4)] |
| 774 | 400 | COD require for 105mg N/L and 15mg P/L | mg COD/L | [[[4]](#endnote-5)] |

**3. Energy transformation happened in the cell and to the environment**

The whole energy pathway roughly includes energy for work (biological activities such as phosphorus transport, etc. and cell proliferation such as poly-P synthesis, glycogen regeneration, etc.) and heat output associated with cell maintenance during the P uptake phase according to the Delft mode. This information has been inserted into the revised manuscript, page 4, lines 69-75. The figure has been added to the supplementary material

Energy

Biological activities

Cell proliferation

Heat output associated

with cell maintenance

Carbon source

Fig. 6. Energy transformation happened in the cell and to the environment

**4. Heat output in different phases of the two sludge samples**

Table 3 Heat output in different phases of the two sludge samples (kJ/mol e-)

|  | H p-release (±SD) | | H p-uptake (±SD) |
| --- | --- | --- | --- |
| CPR sludge | 20.59±3.04 |  | 35.08±12.44 |
| DPR sludge | 45.79±3.41 |  | 84.09±9.46 |

1. T. Kuba, E. Murnleitner, M C. M. Van Loosdercht, J. J. Heijnen. A metabolic model for biological phosphorus removal by denitrifying organisms. Biotechnol. Bioeng. 1996, 52:685-695. [↑](#endnote-ref-2)
2. G. J. F. Smolders, J. van der Mej, M C. M. Van Loosdercht, J. J. Heijnen. Stoichiometric model of the aerobic meatabolism of the aerobic metabolism of the biological phosphorus process. Biotechnol. Bioeng. 1994, 44:837-848. [↑](#endnote-ref-3)
3. T. Kuba, E. Murnleitner, M C. M. Van Loosdercht, J. J. Heijnen. A metabolic model for biological phosphorus removal by denitrifying organisms. Biotechnol. Bioeng. 1996, 52:685-695. [↑](#endnote-ref-4)
4. T. Kuba, M C. M. Van Loosdercht, J. J. Heijnen. Phosphorus and nitrogen removal with minimal COD requirement by integration of denitrifying dephosphatation and nitrification in a two-sludge system. Wat. Res. 1996, 30(7):1702-1710. [↑](#endnote-ref-5)
